# Metabolic Syndrome Alters the Cargo of Mitochondria-Related microRNAs in Swine Mesenchymal Stem Cell-Derived Extracellular Vesicles, Impairing Their Capacity to Repair the Stenotic Kidney

**DOI:** 10.1155/2020/8845635

**Published:** 2020-11-17

**Authors:** Rahele A. Farahani, Xiang-Yang Zhu, Hui Tang, Kyra L. Jordan, Amir Lerman, Lilach O. Lerman, Alfonso Eirin

**Affiliations:** ^1^Department of Internal Medicine, Division of Nephrology and Hypertension, Mayo Clinic, Rochester, MN, USA; ^2^Department of Cardiovascular Diseases, Mayo Clinic, Rochester, MN, USA

## Abstract

**Background:**

Coexisting metabolic syndrome (MetS) and renal artery stenosis (RAS) are linked to poor renal outcomes. Mesenchymal stem/stromal cell- (MSC-) derived extracellular vesicles (EVs) from lean animals show superior ability to repair the experimental MetS+RAS kidney compared to EVs from MetS pig MSCs. We hypothesized that MetS leads to selective packaging in porcine EVs of microRNAs capable of targeting mitochondrial genes, interfering with their capacity to repair the MetS+RAS kidney.

**Methods:**

Five groups of pigs (*n* = 7 each) were studied after 16 weeks of diet-induced MetS and RAS (MetS+RAS) and MetS+RAS 4 weeks after a single intrarenal delivery of EVs harvested from allogeneic adipose tissue-derived MSCs isolated from Lean or MetS pigs, and Lean or MetS sham controls. Single-kidney blood flow (RBF) and glomerular filtration rate (GFR) were assessed in vivo with multidetector CT, whereas EV microRNA cargo, renal tubular mitochondrial structure and bioenergetics, and renal injury pathways were assessed ex vivo.

**Results:**

microRNA sequencing revealed 19 dysregulated microRNAs capable of targeting several mitochondrial genes in MetS-EVs versus Lean-EVs. Lean- and MetS-EVs were detected in the stenotic kidney 4 weeks after administration. However, only MetS-EVs failed to improve renal mitochondrial density, structure, and function or attenuate oxidative stress, tubular injury, and fibrosis. Furthermore, Lean-EVs but not MetS-EVs restored RBF and GFR in MetS+RAS.

**Conclusion:**

MetS alters the cargo of mitochondria-related microRNAs in swine MSC-derived EVs, which might impair their capacity to repair the poststenotic kidney in MetS+RAS. These observations may contribute to develop approaches to improve the efficacy of MSC-EVs for patients with MetS.

## 1. Introduction

Renal artery stenosis (RAS) is becoming more frequently identified in patients with chronic kidney disease and may affect almost 7% of adults older than 65 [1] and more than 50% of patients with diffuse atherosclerosis [2]. Patients with RAS tend to develop renovascular hypertension, which is associated with several cardiovascular complications and favor its progression to end-stage renal disease [3], underscoring the need to identify more effective strategies to preserve the structure and function of the stenotic kidney.

Over the last decade, there has been a growing interest in application of cell-based regenerative therapies for renal disease. In particular, adipose tissue-derived mesenchymal stem/stromal cells (MSCs) have demonstrated important regenerative properties in both experimental [4, 5] and clinical [6, 7] RAS. These cells produce substantial amounts of extracellular vesicles (EVs), which carry genetic and protein material capable of activating a reparative program in recipient cells [8]. We have shown in swine RAS that a single intrarenal administration of MSC-derived EVs attenuates renal inflammation and microvascular damage and improves hemodynamics and function beyond a stenotic lesion [9, 10], positioning MSC-derived EVs as an effective noncellular approach for preserving the stenotic kidney.

A significant proportion of patients with RAS presents with metabolic syndrome (MetS), and their coexistence reduces renal clinical benefits and increases progression to dialysis after renal revascularization [11]. MetS can also exert deleterious effects on MSCs and their daughter EVs, altering their cargo and in vitro reparative potency [12–14]. Moreover, we have recently found that delivery of EVs released by MSCs isolated from MetS pigs failed to repair the injured kidney [15]. However, the mechanisms by which MetS impairs the ability of MSCs to repair the damaged kidney remain unknown.

Emerging experimental evidence suggests that mitochondrial abnormalities and dysfunction may be implicated in the pathogenesis of several forms of renal disease, including MetS [16, 17], RAS [18, 19], and their coexistence [20]. MicroRNAs (miRNAs) are important regulators of the mitochondrial function that modulate the expression of mitochondria-related genes [21]. We have shown that MetS alters the miRNA cargo of EVs derived from porcine adipose tissue MSCs, which could impair the efficacy and limit the therapeutic use of autologous MSCs in subjects with MetS [22]. Therefore, in the current study, we hypothesized that MetS alters the cargo of porcine EVs, leading to selective packaging of miRNAs capable of targeting mitochondrial genes, interfering with the capacity of MetS-EVs to repair the MetS+RAS kidney.

## 2. Materials and Methods

**Figure 1:**
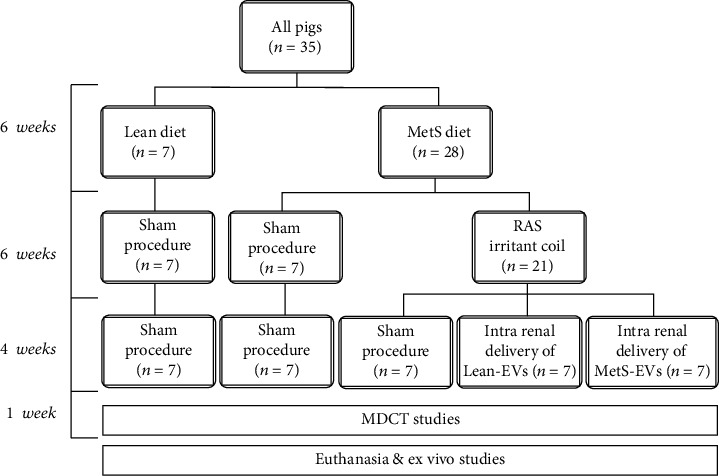
Schematic of the experimental protocol. MetS: metabolic syndrome, RAS: renal artery stenosis, EVs: mesenchymal stem/stromal cell-derived extracellular vesicles, MDCT: multidetector computed tomography. Pigs were studied after 16 weeks of diet-induced MetS and RAS (MetS+RAS), untreated or 4 weeks after intrarenal delivery of EVs isolated from adipose tissue-derived Lean- or MetS-MSCs (*n* = 7 each). Lean and MetS sham served as controls.

We followed the methods of Eirin et al. 2020 [23]. The Mayo Clinic Institutional Animal Care and Use Committee approved all animal experiments. We studied 5 groups of pigs for a total of 16 weeks. Experimental groups included Lean controls, pigs with diet-induced MetS, pigs with MetS plus surgically induced RAS (MetS+RAS), MetS+RAS treated 4 weeks earlier with a Lean-EVs, and MetS+RAS treated with MetS-EVs (*n* = 7 each, Figure 1). At the end of the study, we assessed the single-kidney function in vivo and renal tubular mitochondrial structure and bioenergetics and renal injury pathways ex vivo. In addition, we studied the miRNA cargo of Lean- and MetS-EVs using miRNA sequencing (miRNA-seq).

### 2.1. MSC and EV Isolation, Characterization, and Culture

We isolated EVs from MSCs, which were previously collected from the omental fat tissue from 14 female domestic pigs at the end of the study. Seven pigs (MetS group) were fed a MetS diet consisting of high fat and high fructose (Purina Test Diet, Richmond, IN) [24], whereas 7 Lean pigs (Lean group) were fed regular pig chow (Purina Animal Nutrition). We cultured MSCs in advanced minimal essential medium (Gibco/Invitrogen) with platelet lysate (Mill Creek Life Sciences, LLC, Rochester, MN) [4, 5, 8, 25] and kept them in cell recovery medium. All MSCs expressed MSC markers (CD44+, CD90+, and CD105+ [4, 8] and differentiated into 3 cell linages (adipocytes, osteocytes, and chondrocytes), as we have previously described [4, 5, 8, 25].

We isolated EVs from supernatants of MSCs (10 × 10^6^) by ultracentrifugation, as we have described before [8]. EVs were characterized, and the expression of EV (CD40+, ß1+, CD9+, and CD81+) and MSC (MHC-class-I+ and CD44+) markers confirmed [8, 22, 26]. EVs were then stored at -80°C to study their miRNA, mRNA, and protein cargo, as described below. In addition, Lean- and MetS-EVs were subsequently labeled with PKH26, a red fluorescence dye (Sigma), and injected into MetS+RAS kidneys.

### 2.2. EV microRNA Cargo

We studied the miRNA content of Lean- and MetS-MSC-derived EVs and their parent MSCs using high-throughput miRNA-seq, as we have previously described [8]. We analyzed the miRNA-seq data using the CAP-miRSeq-v workflow [27], a platform that generates aligned BAMs and excel files containing both raw and normalized mature miRNA expression counts. We then utilized the EdgeR to perform differential expression analysis [28, 29] in order to identify miRNAs upregulated (foldchange > 1.4 and *p* ≤ 0.05) and downregulated (foldchange < 0.7 and *p* ≤ 0.05) in MetS-EVs compared to Lean-EVs. Similarly, miRNAs enriched (foldchange > 1.4 and *p* ≤ 0.05) in MetS-EVs compared to MetS-MSCs were identified. For validation purposes, we randomly selected 4 miRNAs (2 upregulated and 2 downregulated in MetS-EVs versus Lean-EVs) and measured their expression by quantitative polymerase chain reaction (qPCR) using RNA U6 small nuclear 6 pseudogene (RNU6B) as normalization/internal control, a common reference gene in miRNA expression studies [30]. We then used TargetScan to identified genes target by these miRNAs and filtered these genes by MitoCarta [31] to identify mitochondria-related genes. Mitochondrial genes were classified using Protein Analysis Through Evolutionary Relationships (PANTHER) [32], a software that classifies genes/proteins by their molecular function, cellular localization, and biological process. Mitochondrial target genes specifically involved in energy pathways were further classified by their primary function by GeneCards® (http://www.genecards.org/), a searchable, integrative database that contains detailed information for all annotated genes.

### 2.3. EV mRNA and Protein Cargo

We explored the gene and protein cargo of Lean- and MetS-MSC-derived EVs with high-throughput mRNA sequencing (mRNA-seq) and liquid chromatography-mass spectrometry (LC-MS/MS) proteomic analysis, as we have previously described [13, 33]. We prepared mRNA-seq libraries using a TruSeq RNA Sample Prep Kit and MSCs sequenced (Illumina). For data analysis, we used the MAPRSeq system, TopHat [34, 35], and featureCounts [36]. mRNA data was normalized and expressed as reads per kilobasepair per million mapped reads. For LC-MS/MS proteomic analysis, EV pellets were solubilized and lysed, and protein samples were denatured. Aliquots were then resolubilized, and samples electrophoresed in gel sections were digested with trypsin [37]. Then, peptides were extracted and transferred onto a PicoFrit and subsequently self-packed with the Dionex UltiMate system (Thermo-Fisher). Following peptide separation and elution, we analyzed them with a mass spectrometer (QExactive, Thermo-Fisher). Differentially expressed proteins (Lean-EVs versus MetS-EVs) were identified, quality confirmed with MaxQuant [38], and differential *p* values adjusted [39]. Mitochondria-related target mRNAs and proteins were identified with MitoCarta [31] and classified by their primary function using Gene Sets Enrichment Analysis (GSEA, https://www.gsea-msigdb.org/gsea/index.jsp).

### 2.4. In Vivo Studies

Additional pigs were placed on a MetS diet for 16 weeks (*n* = 21). Six weeks after diet initiation, animals were anesthetized using intramuscular tiletamine hydrochloride and zolazepam hydrochloride and xylazine (Telazol®, 0.25 g and 0.5 g, respectively). Ketamine (0.2 mg/kg/min IV) and xylazine (0.03 mg/kg/min) were the drugs of choice to maintained anesthesia. Unilateral RAS was induced in all animals by placing a balloon catheter in the right renal artery with the help of fluoroscopy. This catheter was wrapped with an irritant coil that generated gradual narrowing of the renal artery, as well as hypertension in 1-2 weeks [40]. A sham renal angiography was performed in Lean and MetS pigs.

Six weeks following RAS induction, pigs were anesthetized in a similar manner with Telazol®. We then performed a renal angiography to confirm that a significant degree of stenosis was achieved in RAS pigs. Then, we treated MetS+RAS pigs with a single infusion of allogeneic Lean- or MetS-EVs (1 × 10^11^, approximately 100 *μ*g of protein) [9, 41], or vehicle, into the stenotic kidney. EV delivery occurred over 5 min using a 5F catheter positioned in an area of the renal artery proximal to the stenosis (*n* = 7 each). The dose of EVs is based on previous studies from our group in swine MetS+RAS which showed that systemic delivery of EVs was not associated with any side effects or complications and exerted important renoprotective effects [9, 23, 41]. Lean and MetS pigs (*n* = 7 each) underwent only sham procedures (renal angiography plus saline infusion).

Four weeks after treatment with Lean- or MetS-EVs, we collected blood samples from the inferior vena cava to measure total and low-density lipoprotein (LDL) cholesterol, triglyceride, fasting glucose, and insulin levels. To calculate insulin resistance, we utilized the homeostasis model assessment of insulin resistance (HOMA-IR) [24]. Then, renal volume, renal blood flow (RBF), and glomerular filtration rate (GRF) in the single kidney were determined by multidetector computed tomography (MDCT). During MDCT studies, we monitored blood pressure with an intraarterial catheter.

One or two days after later, we euthanized all pigs with intravenous sodium pentobarbital (100 mg/kg, Sleepaway, Fort Dodge) [42]. The kidneys were removed and dissected, and sections were preserved in liquid nitrogen and maintained at -80°C for ex vivo studies, whereas few kidney sections were preserved in formalin or Trump's fixative for staining and electron microscopy studies, respectively.

### 2.5. Renal Function

Renal hemodynamics and function in the single kidney were assessed with a Somatom Sensation-128 MDCT scanner (Siemens Medical Solution, Forchheim, Germany), as we have previously described [43–47]. We injected a central venous bolus of iopamidol (0.5 ml/kg) and performed 140 consecutive scans (330 ms each). Using the Analyze™ (Biomedical Imaging Resource, Mayo Clinic, Rochester, MN) imaging software, we traced regions of interest in the cortex and medulla, which were used to calculate single-kidney regional perfusion (MATLAB, MathWorks). Single-kidney volume (planimetric methods), RBF, and GFR were calculated as we have previously described [48].

### 2.6. EV Tracking

To explore EV retention and localization, we stained stenotic kidney sections with the tubular marker cytokeratin (Abcam, cat# ab7753), as previously described [9, 41]. Labeled (PKH26) EVs in renal sections were counted manually under fluorescence microscopy. The total area of each cross-section was calculated with an image-analysis program (ZEN®, Carl ZEISS SMT; Oberkochen, Germany). The number of EVs/square millimeter was averaged and multiplied by the section thickness and then by the total renal volume (obtained by MDCT). This value (total number of EVs/kidney) was then divided by the number of injected EVs to estimate the retention rate.

### 2.7. Renal Mitochondrial Structure and Function

Immunofluorescence staining with the mitochondrial outer membrane marker preprotein translocases of the outer membrane (TOM)-20 (Santa-Cruz, catalog#: sc-11415, Dallas, TX) was performed to assess the renal mitochondrial density. We took 15-20 images from random fields and quantified them using ZEN®. The renal mitochondrial structure was assessed using a digital electron microscopy (Phillips CM10) at the Mayo Clinic Electron Microscopy Core. Stenotic kidney sections were preserved in Trump's fixative, mounted on mesh grids, and stained with aqueous uranyl acetate and lead citrate. Ten representative tubular cells from 10 different tubules were randomly selected for analysis. The tubular cell mitochondrial area and matrix density were measured in 10 representative mitochondria in these cells using the National Institutes of Health software ImageJ (Version 1.5) [49].

The mitochondrial function was assessed in isolated mitochondria (MITO-ISO kit, ScienCell, cat#: 8268, Carlsbad, CA) [50]. Hydrogen-peroxide (H_2_0_2_) production, cytochrome-c oxidase (COX)-IV activity, and ATP/ADP levels were calculated by colorimetric methods (OxisResearch, BIOXYTECH® H_2_0_2_-560™ Assay, cat# 21024, and abcam, cat# ab83355, Cambridge, United Kingdom, respectively) [51].

### 2.8. Renal Injury Pathways

Production of superoxide anion was assessed in stenotic kidneys by immunofluorescence microscopy using dihydroethidium (DHE) [52]. Tubular injury was assessed in renal cross-sections stained with periodic acid-Schiff (PAS) and scored from 1-5, as previously described [18]. Tubulointerstitial fibrosis was assessed in Masson's trichrome-stained slides and quantified using ZEN® [52].

### 2.9. Statistical Methods

Statistical analysis was performed using JMP 14.1 (SAS, Cary, NC). We used the Shapiro–Wilk test to detect deviations from normality. Results were expressed as mean + SD for normally distributed data, but as median (interquartile range) for nonnormally distributed data. We used parametric (ANOVA/Student's *t*-test with Tukey's post hoc test) and nonparametric (Wilcoxon/Kruskal-Wallis with Steel-Dwass post hoc test) tests as appropriate. Regressions were calculated by the least-squares fit to compare renal mitochondrial damage and poststenotic injury. All tests were two-tailed, and *p* ≤ 0.05 was considered statistically significant.

## 3. Results

The systemic characteristics of all pigs at 16 weeks are presented in Table 1. As expected, body weight and blood pressure were comparably higher in all MetS groups compared to Lean. MetS+RAS, MetS+RAS+Lean-EVs, and MetS+RAS+MetS-EVs showed moderate, but significant and comparable stenoses (*p* > 0.05, ANOVA). MetS pigs also developed hyperlipidemia, reflected in increased total and LDL cholesterol, and triglyceride levels. Although fasting glucose levels were similar among the groups, fasting insulin and HOMA-IR levels were comparably higher in all MetS groups, indicating early prediabetic MetS [24].

### 3.1. MetS Altered the miRNA Cargo of EVs

**Figure 2:**
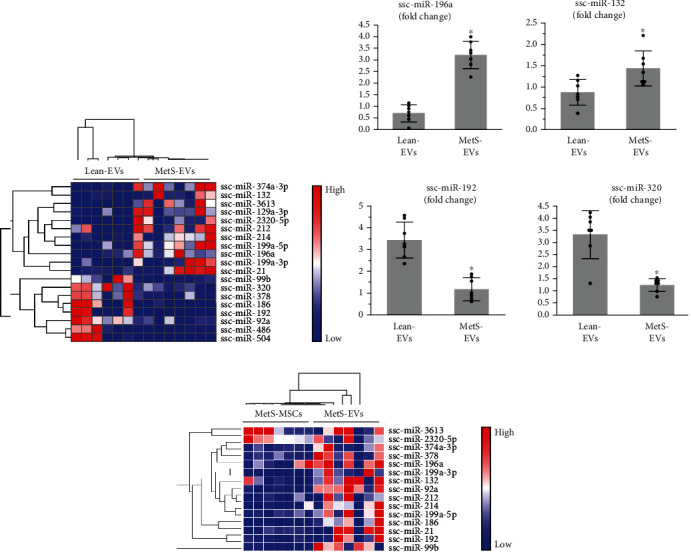
MetS alters the miRNA cargo of MSC-derived EVs. (a) heat map of 11 miRNAs upregulated and 8 miRNAs downregulated in MetS-EVs versus Lean-EVs. (b) Expression of selected differentially expressed candidate miRNAs demonstrated agreement with the miRNA-sequencing analysis, where miR-196a and miR-132 were upregulated and miR-192 and miR-320 downregulated in MetS-EVs. (c) Heat map of 15 out of 19 miRNAs differentially expressed in MetS-EVs versus Lean-EVs that were enriched in MetS-EVs compared to MetS-MSCs. ^∗^*p* < 0.05 vs. Lean-EVs.

We compared the miRNA content of Lean- and MetS-EVs using miRNA sequencing analysis, which identified 11 miRNAs upregulated and 8 miRNAs downregulated in MetS-EVs versus Lean-EVs (Figure 2(a)). qPCR analysis revealed that the expression of randomly selected differentially expressed candidate miRNAs agreed with miRNA-seq analysis, with miR-196a and miR-132 upregulated and miR-192 and miR-320 downregulated in MetS-EVs (Figure 2(b)). Importantly, 15 out of 19 (92.1%) miRNAs differentially expressed in MetS-EVs versus Lean-EVs were enriched in MetS-EVs compared to MetS-MSCs (Figure 2(c)).

### 3.2. miRNAs Dysregulated in MetS-EV Target Mitochondria-Related Genes

**Figure 3:**
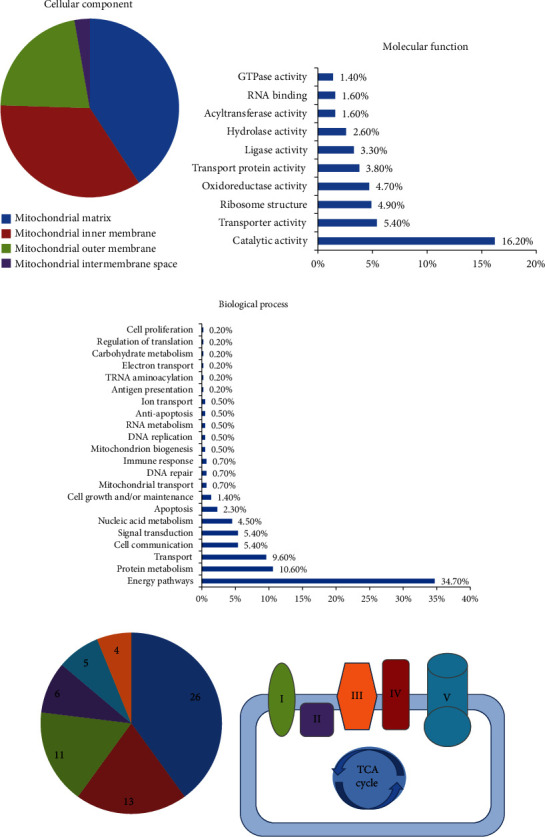
miRNAs dysregulated in MetS-EVs are capable of targeting mitochondria-related genes. Predicted mitochondria target genes of differentially expressed miRNAs were identified (MitoCarta2.0) and classified by their cellular component (a), molecular function (b), and biological process (c). miRNAs dysregulated in MetS-EVs are capable of targeting several genes implicated in mitochondrial energy pathways, including the electron transport chain complexes I, II, III, IV, and V and the tricarboxylic acid (TCA) cycle.

We then performed target analysis of miRNAs differentially expressed in MetS-EVs and found that these miRNAs are capable of targeting a total of 433 mitochondria-related genes (Table S1) primarily located in the mitochondrial matrix and inner mitochondrial membrane (Figure 3(a)). Mitochondrial proteins encoded by these genes have catalytic activity (16.2%), followed by transporter, ribosome, oxidoreductase, transport protein activity, and among others (Figure 3(b)). Importantly, miRNAs dysregulated in MetS-EVs can target genes primarily implicated in mitochondrial energy pathways (37.7%), followed by protein metabolism, transport, cell communication, signal transduction, and others (Figure 3(c)). Specifically, mitochondria-related gene targets implicated in energy pathways included the tricarboxylic acid (TCA) cycle, as well as the electron transport chain (ETC) complexes I-V (Figure 3(d)).

### 3.3. MetS Altered the Expression of Mitochondria-Related Genes and Proteins of EVs

**Figure 4:**
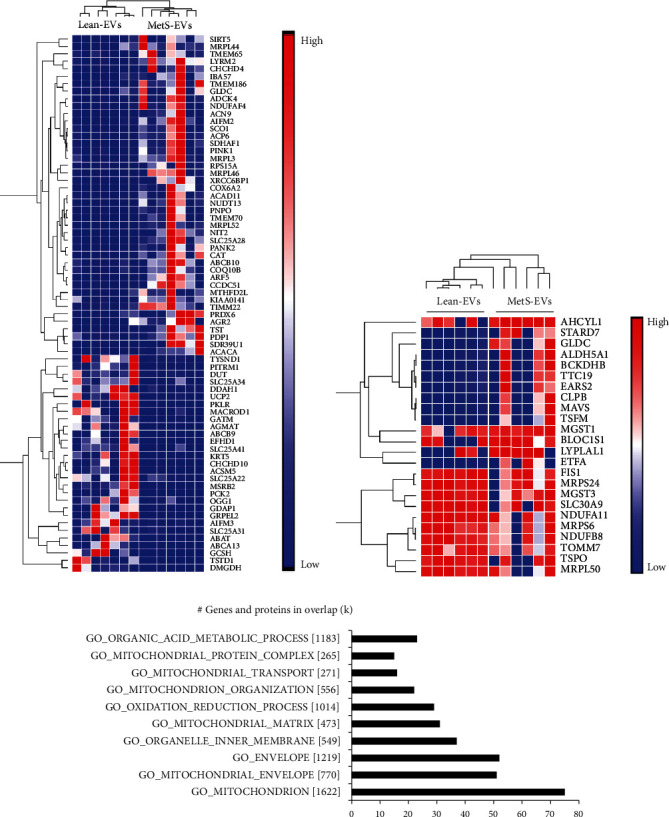
MetS altered the expression of mitochondria-related genes and proteins of EVs. (a) Heat map of 43 mRNAs upregulated and 29 mRNAs downregulated in MetS-EVs versus Lean-EVs. (b) Heat map of 14 proteins upregulated and 10 proteins downregulated in MetS-EVs versus Lean-EVs. (c) Functional analysis of genes and proteins dysregulated in MetS-EVs revealed that they are implicated in important mitochondrial functions, such as organization, transport, and oxidation reduction processes.

In addition, we compared the gene and protein cargo of Lean- and MetS-EVs using mRNA-seq and LC-MS/MS proteomic analysis. mRNA-seq identified a total of 738 mitochondria-related genes, of which 43 were upregulated and 29 downregulated in MetS-EVs versus Lean-EVs (Figure 4(a)). Proteomic analysis identified 663 mitochondrial proteins, of which 14 were upregulated and 10 downregulated in MetS-EVs versus Lean-EVs (Figure 4(b)). Functional analysis of genes and proteins dysregulated in MetS-EVs revealed that they are implicated in important mitochondrial functions, such as organization, transport, and oxidation reduction processes (Figure 4(c)).

### 3.4. EVs Were Retained in the Stenotic Kidney

**Figure 5:**
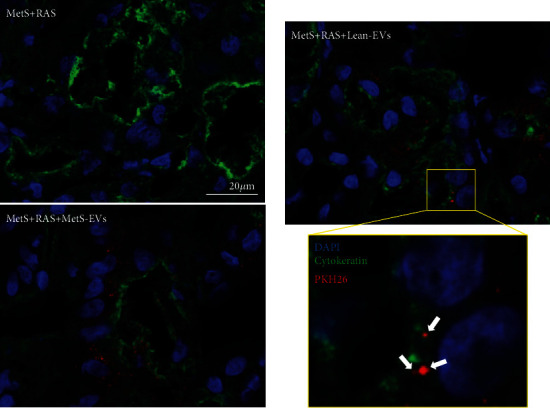
MSC-derived EVs were retained in the poststenotic kidney. Fragments of red immunofluorescent stained EVs (PKH26, arrows; original magnification ×40) were detected in the stenotic kidney of MetS+RAS+Lean-EVs and MetS+RAS+MetS-EVs 4 weeks after administration, but not in untreated MetS+RAS stenotic kidneys. Immunofluorescent costaining with cytokeratin identified EV fragments in the vicinity of renal tubular cells, whereas some of them colocalized with renal tubular cells.

To explore whether MetS impaired the reparative capacity of EVs, we treated MetS+RAS pigs with a single injection of Lean- and MetS-EVs. Four weeks after the intraarterial administration, 2-3% of injected both Lean- and MetS EVs were retained in the stenotic kidney. Immunofluorescence cytokeratin staining similarly identified EV clusters in the tubulointerstitium of Lean-EV and MetS-EV-treated kidneys, some of which colocalized with renal tubular cells (Figure 5).

### 3.5. MetS-EVs Failed to the Improve Renal Mitochondrial Structure and Function

**Figure 6:**
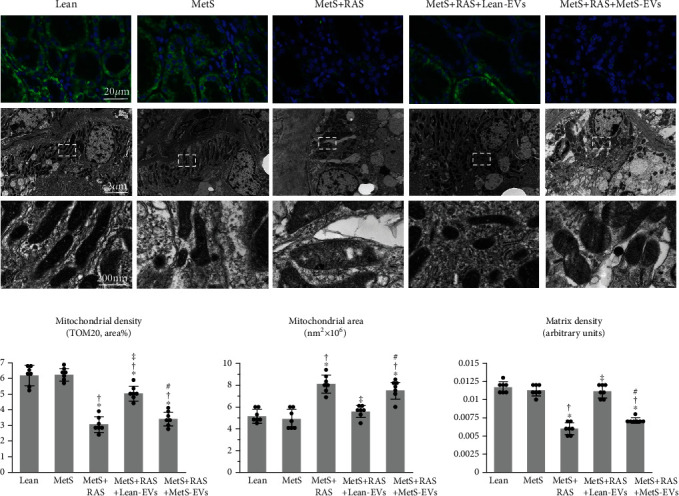
MetS-EVs failed to improve the renal mitochondrial density and structure in MetS+RAS. (a) Representative immunofluorescence staining (original magnification ×40) for the mitochondrial outer membrane marker preprotein translocases of the outer membrane (TOM)-20 (green) and transmission electron microscopy of renal tubular cell mitochondria in study groups. (b) Renal mitochondrial density that decreased in MetS+RAS compared to Lean and MetS, improved in MetS+RAS+Lean-EVs, but failed to improve in MetS+RAS+MetS-EVs, as were the mitochondrial area and matrix density. ^∗^*p* < 0.05 vs. Lean; †*p* < 0.05 vs. MetS; ‡*p* < 0.05 vs. MetS+RAS; #*p* < 0.05 vs. MetS+RAS+Lean-EVs.

Ex vivo studies of EV-treated kidneys showed that the mitochondrial density that decreased in MetS+RAS versus Lean and MetS and increased in MetS+RAS+Lean-EVs failed to improve in MetS+RAS pigs treated with MetS-EVs (Figures 6(a) and 6(b)). Transmission electron microscopy revealed that the renal tubular mitochondrial area increased in MetS+RAS compared to Lean and MetS and was restored to normal levels in MetS+RAS+Lean-EVs, but not in MetS+RAS+MetS-EVs. Contrarily, the mitochondrial matrix density decreased in MetS+RAS versus Lean and MetS, increased in MetS+RAS+Lean-EVs, but not in MetS-EV-treated pigs.

**Figure 7:**
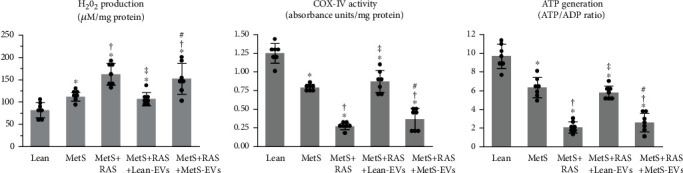
MetS-EVs did not improve the renal mitochondrial function in MetS+RAS. (a) Mitochondrial hydrogen peroxide (H_2_0_2_) that increased in MetS compared to Lean and further increased in MetS+RAS, decreased in MetS+RAS+Lean-EVs, but not in MetS+RAS+MetS-EVs. (b) The cytochrome-c oxidase (COX)-IV activity and ATPADP ratio that decreased in MetS compared to Lean and further decreased in MetS+RAS, increased in MetS+RAS+Lean-EVs, but not in MetS+RAS+MetS-EVs. ^∗^*p* < 0.05 vs. Lean; †*p* < 0.05 vs. MetS; ‡*p* < 0.05 vs. MetS+RAS; #*p* < 0.05 vs. MetS+RAS+Lean-EVs.

Mitochondrial structural abnormalities were associated with functional changes. Production of H_2_0_2_ in isolated mitochondria that was elevated in MetS compared to Lean and further increased in MetS+RAS decreased in MetS+RAS animals treated with Lean-EVs, but not in those treated with MetS-EVs (Figure 7(a)). Mitochondrial energy production, assessed by the COX-IV activity and ATP generation, decreased in MetS compared to Lean, further decreased in MetS+RAS, and increased in MetS+RAS+Lean-EVs. However, MetS-EVs failed to improve either the COX-IV activity or ATP generation.

### 3.6. MetS-EVs Did Not Attenuate Renal Injury and Dysfunction

**Figure 8:**
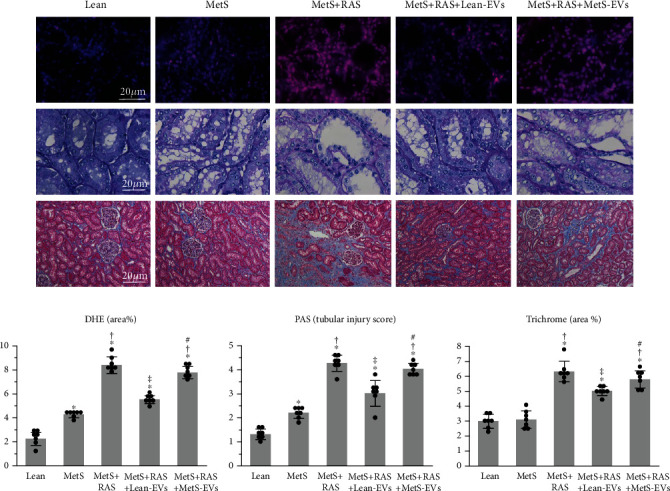
MetS-EVs failed to improve poststenotic kidney injury. (a) Representative kidney dihydroethidium (DHE, red), periodic acid-Schiff (PAS), and trichrome staining in study groups. (b) Renal production of superoxide anion, tubular injury score, and tubulointerstitial fibrosis that increased in MetS+RAS compared to Lean and MetS, decreased in MetS+RAS+Lean-EVs, but not in MetS+RAS+MetS-EVs. ^∗^*p* < 0.05 vs. Lean; †*p* < 0.05 vs. MetS; ‡*p* < 0.05 vs. MetS+RAS; #*p* < 0.05 vs. MetS+RAS+Lean-EVs.

Unlike Lean-EVs, MetS-EVs failed to ameliorate injury and dysfunction in the poststenotic kidney. Renal superoxide anion production and tubular injury observed in MetS compared to Lean further increased in MetS+RAS, decreased in MetS+RAS+Lean-EVs, but failed to decrease in MetS+RAS+MetS-EVs (Figures 8(a) and 8(b)). Likewise, tubulointerstitial fibrosis was markedly higher in MetS+RAS versus Lean and MetS, decreased in MetS+RAS+Lean-EVs, but not in MetS+RAS+MetS-EVs. Notably, single-kidney volume, RBF, and GFR, which were higher in MetS compared to Lean pigs and decreased in MetS+RAS, increased in MetS+RAS+Lean-EVs, but not in MetS+RAS+MetS-EVs (Table 1).

### 3.7. Renal Mitochondrial Damage Correlated with Renal Injury

**Figure 9:**
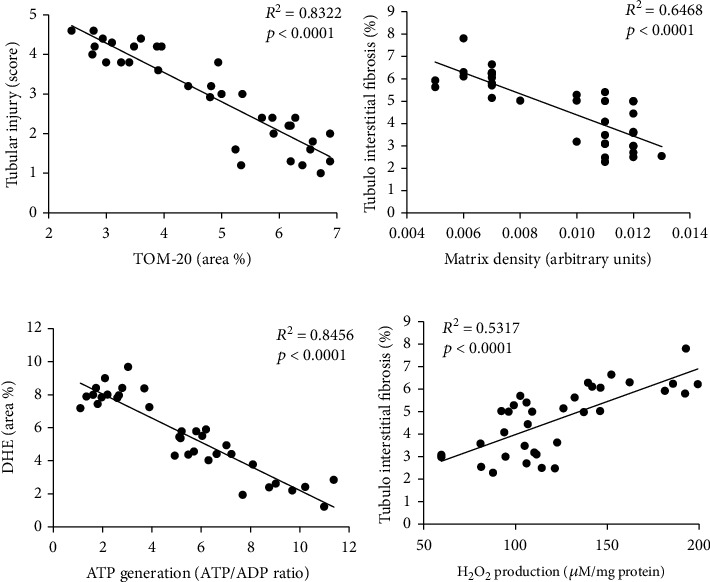
Renal mitochondrial damage correlated with renal injury. (a) The renal mitochondrial density correlated inversely with poststenotic tubular injury. (b) The mitochondrial matrix density correlated inversely with tubulointerstitial fibrosis. (c) Mitochondrial ATP generation correlated inversely with renal production of superoxide anion (DHE). (d) Mitochondrial H_2_0_2_ production correlated directly with tubulointerstitial fibrosis.

Interestingly, we found that the renal mitochondrial density correlated inversely with poststenotic tubular injury (Figure 9(a)). Likewise, the mitochondrial matrix density correlated inversely with tubulointerstitial fibrosis (Figure 9(b)), and mitochondrial ATP generation correlated inversely with renal oxidative stress (Figure 9(c)). Contrarily, mitochondrial H_2_0_2_ production correlated directly with tubulointerstitial fibrosis.

## 4. Discussion

The current study shows that MetS alters the cargo of mitochondria-related miRNAs in swine MSC-derived EVs, which may partly contribute to impair their capacity to repair the poststenotic kidney in MetS+RAS. We interrogated the miRNA expression of porcine Lean- and MetS-EVs using miRNA-seq and found that MetS altered the expression of 19 miRNAs capable of targeting several mitochondrial genes. Intrarenal delivery of Lean-EVs improved renal mitochondrial density, structure, and function and restored the poststenotic kidney function in swine MetS+RAS, whereas MetS-EVs failed to repair them. Importantly, several elements of renal mitochondrial damage correlated well with renal tubulointerstitial injury, suggesting that MetS-induced posttranscriptional modification of mitochondria-related genes may have partly contributed to diminish the potential of MetS-EVs to preserve the stenotic kidney in MetS+RAS. Therefore, our observations may assist in developing novel approaches to improve the therapeutic efficacy of MSC-derived EVs for patients with MetS.

RAS is commonly identified in the elderly population, and its coexistence with MetS hampers benefits of renal revascularization [11]. Recently, MSC-derived EVs emerged as an effective noncellular approach for preserving the poststenotic kidney [53, 54]. The physiological relevance of delivering EVs is supported by previous studies in murine models of kidney injury suggesting that administration of MSC-derived EVs recapitulate the beneficial effect in kidney repair of their parent MSCs [55, 56] and were considered superior to MSCs in some respects [57], suggesting that EVs may confer additional renoprotective effects. We have previously shown that intrarenal delivery of EVs released from adipose tissue-derived MSCs of lean pigs attenuated renal injury in chronic experimental MetS+RAS [12–14], whereas MSC-derived EVs isolated from pigs with MetS failed to decrease stenotic kidney fibrosis or improve GFR [15]. We have also shown that MetS modifies the miRNA cargo of porcine MSC-derived EVs, limiting their efficacy to repair renal tubular cells in vitro [22]. Therefore, we speculated that MetS-induced changes in the miRNA content of EVs might interfere with their ability to repair the ischemic kidney in MetS+RAS.

Renal tubular cells have high content of mitochondria that drive active transport to support the renal tubular function [58] and regulate important cellular functions, including redox status, survival, proliferation, and death. Mitochondrial abnormalities and dysfunction are implicated in the pathogenesis of RAS, as mitoprotection restores the renal function [19] and improves revascularization outcomes in experimental [18] and clinical [59] RAS. In the current study, Lean-EVs exerted important mitoprotective properties in the stenotic kidney. The renal mitochondrial density that was reduced in MetS+RAS improved 4 weeks after intrarenal delivery of Lean-EVs. The renal mitochondrial area increased in MetS+RAS, likely reflecting mitochondrial swelling, but was restored in Lean-EV-treated pigs. Likely because mitochondrial swelling disrupts cristae shape [60], the mitochondrial matrix density that decreased in MetS+RAS was normalized in MetS+RAS pigs treated with Lean-EVs.

Mitochondrial cristae membranes with embedded ETC enzymes contribute to energy production by providing a large surface area for chemical reactions [61]. Thus, an increase in the cristae surface area promotes mitochondrial respiration. In agreement, we found that Lean-EVs not only improved the mitochondrial structure but also increased the activity of COX-IV, the final stage of the ETC that catalyzes transfer of electrons from cytochrome-c to molecular oxygen [62]. Similarly, ATP generation that was blunted in MetS+RAS increased in Lean-EV-treated pigs, indicating improved mitochondrial energy production.

Mitochondrial structural damage and dysfunction are often accompanied by increased oxidative stress, mainly due to production of superoxide from complexes I and III [63], and in turn, H_2_0_2_, which damages several mitochondrial constituents, creating a vicious cycle of mitochondrial injury and oxidative stress [64]. Indeed, superoxide anion (DHE staining) and H_2_0_2_ production in isolated mitochondria increased in MetS+RAS, but decreased to MetS levels in MetS+RAS+Lean-EVs. Notably, delivery of Lean-EVs also attenuated tubulointerstitial injury and fibrosis and improved stenotic kidney volume, RBF, and GFR. Interestingly, renal mitochondrial damage (density, matrix density, and H_2_0_2_ generation) correlated with renal tubulointerstitial injury (injury score and fibrosis), whereas mitochondrial energy production (ATP generation) correlated inversely with renal oxidative stress (DHE staining). Therefore, our observations support the premise that renal mitochondrial injury is implicated in the pathogenesis of MetS+RAS and underscore the potential of Lean-EVs to preserve the poststenotic kidney.

Contrarily, MetS-EVs failed to preserve mitochondria or improve the stenotic kidney function. Unlike Lean-EVs, MetS-EVs did not improve the renal mitochondrial density, area, or matrix density, did not decrease mitochondrial H_2_0_2_ generation, or increase COX-IV activity and ATP production. Moreover, MetS-EVs did not attenuate renal oxidative stress, tubular injury, or tubulointerstitial fibrosis, or improve RBF or GFR, indicating that MetS limited the capacity of MSC-derived EVs to repair the stenotic kidney.

To explore mechanisms by which MetS failed to improve the mitochondrial structure and function in MetS-EV-treated pigs, we characterized the EV cargo of miRNA, which are important regulators of the mitochondrial function that modulate the expression of mitochondria-related genes [21]. We identified 19 miRNAs dysregulated in MetS-EVs, in agreement with our previous findings [22]. Our results should be interpreted with caution given that differential expressed miRNAs are not among the most abundant in EVs. Nevertheless, most miRNAs differentially expressed in MetS-EVs versus Lean-EVs were enriched in MetS-EVs compared to their parent MSCs, suggesting that a significant proportion of these miRNAs was selectively packed in MSC-derived EVs. Classification of their mitochondrial targets revealed that these proteins are primarily located in the mitochondrial matrix and inner membranes, where principal components of aerobic respiration take place. Subsequent functional analysis showed that they are primarily implicated in energy pathways, including the TCA cycle and ETC. Therefore, MetS-induced changes in the miRNA profile of EVs might have exerted important posttranscriptional changes in mitochondria-related genes in recipient cells, which might have contributed to mitochondrial injury and in turn, impaired the capacity of MetS-EVs to repair the stenotic kidney. Importantly, our findings are in agreement with our recent observation that MetS alters the miRNA cargo of human MSC-derived EVs [65], suggesting that MetS-induced changes in the content of EVs are conserved in human subjects. Therefore, miRNAs packed in MSC-derived EVs may have important implications for the use of autologous EVs as a therapeutic regenerative strategy.

Remarkably, we found that diet-induced MetS also altered the expression of mitochondria-related genes and proteins in swine MSC-derived EVs, which were involved in important mitochondrial functions, such as organization, transport, and oxidation reduction processes. Therefore, MetS-induced changes in the mRNA, miRNA, and protein cargo of EVs could have interfered with the capacity of EVs to preserve renal mitochondria and repair the poststenotic kidney in MetS+RAS.

Our study is limited by the short duration of the disease and the use of relatively young pigs. However, our model recapitulates the synergistic interaction between human RAS and MetS, and 16 weeks of high-fat/fructose diet sufficed to alter the miRNA cargo of MSC-derived EVs. The cargo of cultured MSC-derived EVs might differ from the content of EVs secreted by MSCs in vivo. Isolation and culture conditions may affect the behavior of MSCs and their daughter EVs, making their in vivo activity difficult to predict [66]. Our previous studies have shown that retention of EVs in the stenotic kidney peaked at 2 days and decreased thereafter, remaining at 2-3% by 4 weeks after injection [9, 41]. In agreement, we found that four weeks after intrarenal delivery, Lean- and MetS-EVs were similarly detected within the stenotic kidney tubulointerstitium, and some clusters colocalized with tubular cells. Yet, we cannot rule out differences in uptake and cytoplasmic distribution of Lean- and MetS-EVs in recipient cells, which could have modulated their reparative potency. Longer follow-up studies are needed to establish the causal relationship between the miRNA content and reno- and mitoprotective properties of Lean-EVs and explore the extent to which these are conserved across species.

## 5. Conclusions

In summary, our study shows that MetS modifies the miRNA cargo of swine MSC-derived EVs. miRNA targets include several mitochondrial genes encoding for proteins implicated in mitochondrial energy pathways. Delivery of MetS-EVs failed to improve the renal mitochondrial or poststenotic kidney structure and function in MetS+RAS. Renal mitochondrial damage correlated with renal injury, implying that MetS-induced posttranscriptional regulation of mitochondria-related genes could have partly contributed to impair the in vivo reparative capacity of MetS-EVs. Additional studies are needed to test the therapeutic efficacy of Lean- and MetS-MSC-derived EVs in patients with MetS and RAS.

## Figures and Tables

**Table 1:** Systemic characteristics and single-kidney function in study groups at 16 weeks.

	Lean	MetS	MetS+RAS	MetS+RAS+ LeanEVs	MetS+RAS+ MetS-EVs
Body weight (kg)	72.4 ± 7.2	92.0 ± 6.7^∗^	92.4 ± 5.7^∗^	90.4 ± 8.6^∗^	88.4 ± 7.1^∗^
MAP (mmHg)	93.6 ± 4.9	124.2 ± 7.2^∗^	125.1 ± 6.6^∗^	120.6 ± 13.0^∗^	120.6 ± 8.4^∗^
Degree of stenosis (%)	0	0	60.0 ± 13.9^∗^†	65.0 ± 7.6^∗^†	67.5 ± 6.0^∗^†
Total cholesterol (mg/dl)	84.5 (78.0-90.1)	328.0 (315.1-481.1)^∗^	345.2 (321.2-406.2)^∗^	345.2 (323.7-413.1)^∗^	316.5 (310.5-329.5)^∗^
LDL cholesterol (mg/dl)	32.7 ± 5.6	338.5 ± 96.8^∗^	363.8 ± 49.0^∗^	339.8 ± 35.6^∗^	352.4 ± 39.3^∗^
Triglycerides (mg/dl)	7.6 (5.9-8.7)	17.3 (13.0-22.5)^∗^	15.1 (14.2-16.1)^∗^	16.7 (12.9-17.3)^∗^	14.8 (12.4-17.0)^∗^
Fasting glucose (mg/dl)	121.7 ± 5.4	113.5 ± 15.3	115.5 ± 14.1	117.8 ± 18.4	121.8 ± 17.2
Fasting insulin (*μ*U/ml)	0.4 (0.4-0.5)	0.8 (0.7-0.8)^∗^	0.8 (0.7-0.8)^∗^	0.8 (0.7-0.8)^∗^	0.7 (0.7-0.8)^∗^
HOMA-IR score	0.6 (0.5-0.7)	1.9 (1.5-1.9)^∗^	1.8 (1.7-1.8)^∗^	1.7 (1.7-1.8)^∗^	1.8 (1.5-1.9)^∗^
Renal volume (ml)	131.9 ± 13.7	217.3 ± 11.8^∗^	185.0 ± 19.0^∗^†	229.5 ± 47.3^∗^‡	168.5 ± 25.1^∗^†#
RBF (ml/min)	502.0 ± 69.3	811.6 ± 78.9^∗^	615.4 ± 74.4^∗^†	840.3 ± 241.6^∗^‡	660.3 ± 67.6^∗^†#
GFR (ml/min)	74.8 ± 9.7	138.9 ± 21.2^∗^	97.2 ± 11.9^∗^†	128.0 ± 33.3^∗^‡	93.8 ± 13.8^∗^†#

MAP: mean arterial pressure; LDL: low-density lipoprotein; HOMA-IR: homeostasis model assessment of insulin resistance; RBF: renal blood flow; GFR: glomerular filtration rate. ^∗^*p* < 0.05 vs. Lean; †*p* < 0.05 vs. MetS; ‡*p* < 0.05 vs. MetS + RAS; #*p* < 0.05 vs. MetS+RAS+Lean-EVs.

## Data Availability

The miRNA-seq data of each individual sample generated in this study are available online at the following link: https://figshare.com/articles/miRNA-seq_data_Lean-EVs_and_MetS-EVs/12534458.

## Supplementary Materials

Supplementary Materials Table S1: mitochondria-related targets of miRNAs dysregulated in MetS-EVs.

## References

[1] Hansen K. J., Edwards M. S., Craven T. E. (2002). Prevalence of renovascular disease in the elderly: a population-based study. *Journal of Vascular Surgery*.

[2] Uzu T., Takeji M., Yamada N. (2002). Prevalence and outcome of renal artery stenosis in atherosclerotic patients with renal dysfunction. *Hypertension Research*.

[3] Textor S. C., Lerman L. O. (2015). Paradigm shifts in atherosclerotic renovascular disease: where are we now?. *Journal of the American Society of Nephrology*.

[4] Eirin A., Zhu X. Y., Krier J. D. (2012). Adipose tissue-derived mesenchymal stem cells improve revascularization outcomes to restore renal function in swine atherosclerotic renal artery stenosis. *Stem Cells*.

[5] Eirin A., Zhang X., Zhu X. Y. (2014). Renal vein cytokine release as an index of renal parenchymal inflammation in chronic experimental renal artery stenosis. *Nephrology, Dialysis, Transplantation*.

[6] Saad A., Dietz A. B., Herrmann S. M. S. (2017). Autologous mesenchymal stem cells increase cortical perfusion in renovascular disease. *Journal of the American Society of Nephrology*.

[7] Abumoawad A., Saad A., Ferguson C. M. (2020). In a phase 1a escalating clinical trial, autologous mesenchymal stem cell infusion for renovascular disease increases blood flow and the glomerular filtration rate while reducing inflammatory biomarkers and blood pressure. *Kidney International*.

[8] Eirin A., Riester S. M., Zhu X. Y. (2014). MicroRNA and mRNA cargo of extracellular vesicles from porcine adipose tissue-derived mesenchymal stem cells. *Gene*.

[9] Eirin A., Zhu X. Y., Puranik A. S. (2017). Mesenchymal stem cell-derived extracellular vesicles attenuate kidney inflammation. *Kidney International*.

[10] Eirin A., Zhu X. Y., Ebrahimi B. (2015). Intrarenal delivery of mesenchymal stem cells and endothelial progenitor cells attenuates hypertensive cardiomyopathy in experimental renovascular hypertension. *Cell Transplantation*.

[11] Davies M. G., Saad W. E., Bismuth J., Naoum J. J., Peden E. K., Lumsden A. B. (2010). Impact of metabolic syndrome on the outcomes of percutaneous renal angioplasty and stenting. *Journal of Vascular Surgery*.

[12] Eirin A., Lerman L. O. (2019). Stem Cell-Derived Extracellular Vesicles for Renal Repair: Do Cardiovascular Comorbidities Matter?. *American Journal of Physiology-Renal Physiology*.

[13] Eirin A., Zhu X. Y., Woollard J. R. (2019). Metabolic syndrome interferes with packaging of proteins within porcine mesenchymal stem cell-derived extracellular vesicles. *Stem Cells Translational Medicine*.

[14] Pawar A. S., Eirin A., Krier J. D. (2019). Alterations in genetic and protein content of swine adipose tissue-derived mesenchymal stem cells in the metabolic syndrome. *Stem Cell Research*.

[15] Song T., Eirin A., Zhu X. (2020). Mesenchymal stem cell-derived extracellular vesicles induce regulatory T cells to ameliorate chronic kidney injury. *Hypertension*.

[16] Eirin A., Woollard J. R., Ferguson C. M. (2017). The metabolic syndrome induces early changes in the swine renal medullary mitochondria. *Translational Research*.

[17] Eirin A., Hedayat A. F., Ferguson C. M., Textor S. C., Lerman A., Lerman L. O. (2018). Mitoprotection preserves the renal vasculature in porcine metabolic syndrome. *Experimental Physiology*.

[18] Eirin A., Li Z., Zhang X. (2012). A mitochondrial permeability transition pore inhibitor improves renal outcomes after revascularization in experimental atherosclerotic renal artery stenosis. *Hypertension*.

[19] Eirin A., Ebrahimi B., Zhang X. (2014). Mitochondrial protection restores renal function in swine atherosclerotic renovascular disease. *Cardiovascular Research*.

[20] Nargesi A. A., Zhang L., Tang H. (2019). Coexisting renal artery stenosis and metabolic syndrome magnifies mitochondrial damage, aggravating poststenotic kidney injury in pigs. *Journal of Hypertension*.

[21] Borralho P. M., Rodrigues C. M., Steer C. J. (2015). microRNAs in mitochondria: an unexplored niche. *Advances in Experimental Medicine and Biology*.

[22] Meng Y., Eirin A., Zhu X. Y. (2018). The metabolic syndrome alters the miRNA signature of porcine adipose tissue-derived mesenchymal stem cells. *Cytometry. Part A*.

[23] Eirin A., Ferguson C. M., Zhu X. Y. (2020). Extracellular vesicles released by adipose tissue-derived mesenchymal stromal/stem cells from obese pigs fail to repair the injured kidney. *Stem Cell Research*.

[24] Pawar A. S., Zhu X. Y., Eirin A. (2015). Adipose tissue remodeling in a novel domestic porcine model of diet-induced obesity. *Obesity*.

[25] Ebrahimi B., Eirin A., Li Z. (2013). Mesenchymal stem cells improve medullary inflammation and fibrosis after revascularization of swine atherosclerotic renal artery stenosis. *PLoS One*.

[26] Eirin A., Zhu X.-Y., Puranik A. S. (2016). Comparative proteomic analysis of extracellular vesicles isolated from porcine adipose tissue-derived mesenchymal stem/stromal cells. *Scientific Reports*.

[27] Sun Z., Evans J., Bhagwate A. (2014). CAP-miRSeq: a comprehensive analysis pipeline for microRNA sequencing data. *BMC Genomics*.

[28] Dudakovic A., Camilleri E., Riester S. M. (2014). High-resolution molecular validation of self-renewal and spontaneous differentiation in clinical-grade adipose-tissue derived human mesenchymal stem cells. *Journal of Cellular Biochemistry*.

[29] Robinson M. D., McCarthy D. J., Smyth G. K. (2009). edgeR: a bioconductor package for differential expression analysis of digital gene expression data. *Bioinformatics*.

[30] Kaija H., Pakanen L., Porvari K. (2020). RNU6B, a frequent reference in miRNA expression studies, differentiates between deaths caused by hypothermia and chronic cardiac ischemia. *International Journal of Legal Medicine*.

[31] Calvo S. E., Clauser K. R., Mootha V. K. (2016). MitoCarta2.0: an updated inventory of mammalian mitochondrial proteins. *Nucleic Acids Research*.

[32] Mi H., Lazareva-Ulitsky B., Loo R. (2005). The PANTHER database of protein families, subfamilies, functions and pathways. *Nucleic Acids Research*.

[33] Meng Y., Eirin A., Zhu X. Y. (2018). The metabolic syndrome modifies the mRNA expression profile of extracellular vesicles derived from porcine mesenchymal stem cells. *Diabetology and Metabolic Syndrome*.

[34] Kalari K. R., Nair A. A., Bhavsar J. D. (2014). MAP-RSeq: mayo analysis pipeline for RNA sequencing. *BMC Bioinformatics*.

[35] Kim D., Pertea G., Trapnell C., Pimentel H., Kelley R., Salzberg S. L. (2013). TopHat2: accurate alignment of transcriptomes in the presence of insertions, deletions and gene fusions. *Genome Biology*.

[36] Liao Y., Smyth G. K., Shi W. (2014). featureCounts: an efficient general purpose program for assigning sequence reads to genomic features. *Bioinformatics*.

[37] Hogan M. C., Bakeberg J. L., Gainullin V. G. (2015). Identification of biomarkers for PKD1 using urinary exosomes. *Journal of the American Society of Nephrology*.

[38] Cox J., Hein M. Y., Luber C. A., Paron I., Nagaraj N., Mann M. (2014). Accurate proteome-wide label-free quantification by delayed normalization and maximal peptide ratio extraction, termed MaxLFQ. *Molecular & Cellular Proteomics*.

[39] Kim K. I., van de Wiel M. A. (2008). Effects of dependence in high-dimensional multiple testing problems. *BMC Bioinformatics*.

[40] Lerman L. O., Schwartz R. S., Grande J. P., Sheedy P. F., Romero J. C. (1999). Noninvasive evaluation of a novel swine model of renal artery stenosis. *Journal of the American Society of Nephrology*.

[41] Eirin A., Zhu X. Y., Jonnada S., Lerman A., van Wijnen A. J., Lerman L. O. (2018). Mesenchymal stem cell-derived extracellular vesicles improve the renal microvasculature in metabolic renovascular disease in swine. *Cell Transplantation*.

[42] Eirin A., Williams B. J., Ebrahimi B. (2014). Mitochondrial targeted peptides attenuate residual myocardial damage after reversal of experimental renovascular hypertension. *Journal of Hypertension*.

[43] Ferguson C. M., Eirin A., Michalak G. J. (2018). Intrarenal fat deposition does not interfere with the measurement of single-kidney perfusion in obese swine using multi-detector computed tomography. *Journal of Cardiovascular Computed Tomography*.

[44] Ferguson C. M., Eirin A., Michalak G. J. (2019). Renal adiposity does not preclude quantitative assessment of renal function using dual-energy multidetector CT in mildly obese human subjects. *Academic Radiology*.

[45] Eirin A., Ebrahimi B., Zhang X. (2012). Changes in glomerular filtration rate after renal revascularization correlate with microvascular hemodynamics and inflammation in swine renal artery stenosis. *Circulation. Cardiovascular Interventions*.

[46] Chade A. R., Zhu X., Lavi R. (2009). Endothelial progenitor cells restore renal function in chronic experimental renovascular disease. *Circulation*.

[47] Zhu X.-Y., Chade A. R., Rodriguez-Porcel M. (2004). Cortical microvascular remodeling in the stenotic kidney. *Arteriosclerosis, Thrombosis, and Vascular Biology*.

[48] Krier J. D., Ritman E. L., Bajzer Z., Romero J. C., Lerman A., Lerman L. O. (2001). Noninvasive measurement of concurrent single-kidney perfusion, glomerular filtration, and tubular function. *American Journal of Physiology. Renal Physiology*.

[49] Schneider C. A., Rasband W. S., Eliceiri K. W. (2012). NIH image to ImageJ: 25 years of image analysis. *Nature Methods*.

[50] Zhang X., Li Z. L., Crane J. A. (2014). Valsartan regulates myocardial autophagy and mitochondrial turnover in experimental hypertension. *Hypertension*.

[51] Pi J., Bai Y., Zhang Q. (2007). Reactive oxygen species as a signal in glucose-stimulated insulin secretion. *Diabetes*.

[52] Eirin A., Zhu X. Y., Urbieta-Caceres V. H. (2011). Persistent kidney dysfunction in swine renal artery stenosis correlates with outer cortical microvascular remodeling. *American Journal of Physiology. Renal Physiology*.

[53] Aghajani Nargesi A., Lerman L. O., Eirin A. (2017). Mesenchymal stem cell-derived extracellular vesicles for kidney repair: current status and looming challenges. *Stem Cell Research & Therapy*.

[54] Nargesi A. A., Lerman L. O., Eirin A. (2017). Mesenchymal stem cell-derived extracellular vesicles for renal repair. *Current Gene Therapy*.

[55] Wang R., Lin M., Li L., Li L., Qi G., Rong R. (2014). Bone marrow mesenchymal stem cell-derived exosome protects kidney against ischemia reperfusion injury in rats. *Zhonghua Yi Xue Za Zhi*.

[56] He J., Wang Y., Sun S. (2012). Bone marrow stem cells-derived microvesicles protect against renal injury in the mouse remnant kidney model. *Nephrology*.

[57] He J., Wang Y., Lu X. (2015). Micro-vesicles derived from bone marrow stem cells protect the kidney both in vivo and in vitro by microRNA-dependent repairing. *Nephrology*.

[58] Bhargava P., Schnellmann R. G. (2017). Mitochondrial energetics in the kidney. *Nature Reviews. Nephrology*.

[59] Saad A., Herrmann S. M. S., Eirin A. (2017). Phase 2a clinical trial of mitochondrial protection (Elamipretide) during stent revascularization in patients with atherosclerotic renal artery stenosis. *Circulation. Cardiovascular Interventions*.

[60] Kaasik A., Safiulina D., Zharkovsky A., Veksler V. (2007). Regulation of mitochondrial matrix volume. *American Journal of Physiology. Cell Physiology*.

[61] Leveille C. F., Mikhaeil J. S., Turner K. D., Silvera S., Wilkinson J., Fajardo V. A. (2017). Mitochondrial cristae density: a dynamic entity that is critical for energy production and metabolic power in skeletal muscle. *The Journal of Physiology*.

[62] Li Y., Park J. S., Deng J. H., Bai Y. (2006). Cytochrome c oxidase subunit IV is essential for assembly and respiratory function of the enzyme complex. *Journal of Bioenergetics and Biomembranes*.

[63] Murphy M. P. (2013). Mitochondrial dysfunction indirectly elevates ROS production by the endoplasmic reticulum. *Cell Metabolism*.

[64] Boveris A., Valdez L. B., Zaobornyj T., Bustamante J. (2006). Mitochondrial metabolic states regulate nitric oxide and hydrogen peroxide diffusion to the cytosol. *Biochimica et Biophysica Acta*.

[65] Li Y., Meng Y., Zhu X. (2020). Metabolic syndrome increases senescence-associated micro-RNAs in extracellular vesicles derived from swine and human mesenchymal stem/stromal cells. *Cell Communication and Signaling: CCS*.

[66] Kozlowska U., Krawczenko A., Futoma K. (2019). Similarities and differences between mesenchymal stem/progenitor cells derived from various human tissues. *World Journal of Stem Cells*.

