# Supplementary material for: Metabolic Syndrome Alters the Cargo of Mitochondria-Related microRNAs in Swine Mesenchymal Stem Cell-Derived Extracellular Vesicles, Impairing Their Capacity to Repair the Stenotic Kidney
Source: Stem Cells Int. 2020 Nov 17;2020:8845635. doi: 10.1155/2020/8845635 (PMC7685840; doi:10.1155/2020/8845635)
Supplement: Supplementary Materials — Table S1: mitochondria-related targets of miRNAs dysregulated in MetS-EVs. [file 8845635.f1.zip › Supplementary_Description.docx]

Table S1. Mitochondria-related targets of miRNAs dysregulated in MetS-EVs.
