# Supplementary material for: Metabolic Syndrome Alters the Cargo of Mitochondria-Related microRNAs in Swine Mesenchymal Stem Cell-Derived Extracellular Vesicles, Impairing Their Capacity to Repair the Stenotic Kidney
Source: Stem Cells Int. 2020 Nov 17;2020:8845635. doi: 10.1155/2020/8845635 (PMC7685840; doi:10.1155/2020/8845635)
Supplement: Supplementary Materials — Table S1: mitochondria-related targets of miRNAs dysregulated in MetS-EVs. [file 8845635.f1.zip › Table S1.docx]

*Table S1.*  Mitochondria-related targets of miRNAs dysregulated in MetS-EVs.

| Gene symbol | | |  |  | |  | |  | |  | |  | |  | |  | |  | |
| --- | --- | --- | --- | --- | --- | --- | --- | --- | --- | --- | --- | --- | --- | --- | --- | --- | --- | --- | --- |
| AASS | | ACSL6 | | | ALDH1L2 | | ATPAF1 | | CBR4 | | COA5 | | CYB5B | | DUS2 | | FBXL4 | GPD2 | |
| ABAT | | ACSM2A | | | ALDH2 | | ATXN2 | | CCBL2 | | COQ10B | | CYCS | | DUT | | FECH | GPT2 | |
| ABCB8 | | ACSS1 | | | ALDH5A1 | | BAK1 | | CCDC109B | | COQ5 | | CYP24A1 | | ECHDC2 | | FPGS | GRHPR | |
| ABCB9 | | ADHFE1 | | | ALDH6A1 | | BCAT2 | | CCDC51 | | COQ7 | | DARS2 | | ECHDC3 | | FTSJ2 | GRPEL2 | |
| ABCD2 | | AFG3L2 | | | ALDH7A1 | | BCKDHB | | CCDC58 | | COX15 | | DBT | | EHHADH | | FUNDC2 | GRSF1 | |
| ABCD3 | | AGMAT | | | ALDH9A1 | | BCKDK | | CCDC90B | | COX19 | | DECR1 | | EMC2 | | FXN | GSTK1 | |
| ABCF2 | | AGPAT5 | | | AMACR | | BCL2 | | CHCHD10 | | COX6C | | DHODH | | EPHX2 | | GATC | GTPBP10 | |
| ABHD10 | | AGR2 | | | ANGEL2 | | BCL2L2 | | CHCHD4 | | COX7A2 | | DHRSX | | ETFA | | GDAP1 | GUF1 | |
| ACACB | | AGXT2 | | | APOOL | | BID | | CHDH | | COX7A2L | | DHX30 | | FABP1 | | GFM1 | HEMK1 | |
| ACAD11 | | AHCYL1 | | | ARMC10 | | BNIP3L | | CISD1 | | COX7B | | DLAT | | FAHD1 | | GHITM | HIBADH | |
| ACAD8 | | AIFM1 | | | ASAH2 | | C10orf2 | | CISD2 | | COX7C | | DLD | | FAHD2A | | GK | HIGD1A | |
| ACADSB | | AIFM2 | | | ATAD1 | | C15orf40 | | CLIC4 | | COX8C | | DLST | | FAM136A | | GLRX2 | HINT3 |  |
| ACN9 | | AK3 | | | ATP10D | | C15orf61 | | CLPB | | CPOX | | DMGDH | | FAM162A | | GLUD1 | HK2 |  |
| ACOT13 | | AK4 | | | ATP5G1 | | C19orf52 | | CLPX | | CPS1 | | DNA2 | | FAM213A | | GLYAT | HSDL1 |  |
| ACOT2 | | AKAP1 | | | ATP5G3 | | C2orf69 | | CLYBL | | CPT1A | | DNAJC11 | | FARS2 | | GLYCTK | HSPB7 |  |
| ACOX1 | | AKAP10 | | | ATP5J | | C6orf203 | | CMC1 | | CRYZ | | DNAJC19 | | FASTK | | GNG5 | IDE |  |
| ACP6 | AKR7A2 | | | ATP5J2 | | | CA5B | | CMPK2 | | CS | | DNAJC30 | | FASTKD1 | | GOT2 | IDH1 |  |
| ACSL4 | ALDH1L1 | | | ATP5S | | | CAT | | COA1 | | CYB5A | | DNM1L | | FASTKD2 | | GPAM | IDI1 |  |

|  |  |  | |  | |  | |  | |  | |  | |  | |  | |  |
| --- | --- | --- | --- | --- | --- | --- | --- | --- | --- | --- | --- | --- | --- | --- | --- | --- | --- | --- |
| MAVS | MPV17L | | MRPS23 | | MTRF1L | | NFS1 | | OXA1L | | PDP2 | | PTCD1 | | RNMTL1 | | SLC25A12 | |
| MCUR1 | MRM1 | | MRPS25 | | MUL1 | | NGRN | | OXNAD1 | | PDPR | | PTCD3 | | ROMO1 | | SLC25A16 | |
| ME2 | MRPL11 | | MRPS30 | | MUT | | NIPSNAP3B | | OXR1 | | PGS1 | | PTPMT1 | | RPIA | | SLC25A21 | |
| MECR | MRPL13 | | MRPS36 | | NADK2 | | NIT2 | | PAICS | | PHYH | | PTPN4 | | RPS15A | | SLC25A22 | |
| METTL15 | MRPL17 | | MRPS6 | | NAGS | | NLN | | PAK7 | | PI4KA | | PTRH2 | | SARDH | | SLC25A23 | |
| METTL8 | MRPL18 | | MRRF | | NARS2 | | NME4 | | PANK2 | | PMPCA | | PTS | | SCO1 | | SLC25A24 | |
| MFF | MRPL19 | | MRS2 | | NBR1 | | NME6 | | PARL | | PMPCB | | PXMP4 | | SDHA | | SLC25A25 | |
| MFN1 | MRPL22 | | MSRA | | NCEH1 | | NNT | | PARS2 | | PNPO | | PYCR2 | | SDHC | | SLC25A28 | |
| MGARP | MRPL27 | | MSRB2 | | NCOA4 | | NRD1 | | PCBD2 | | POLDIP2 | | PYURF | | SDHD | | SLC25A32 | |
| MGST3 | MRPL3 | | MSRB3 | | NDUFA5 | | NSUN3 | | PCCB | | POLG | | QRSL1 | | SERAC1 | | SLC25A33 | |
| MICU1 | MRPL30 | | MTCH2 | | NDUFAF4 | | NSUN4 | | PCK2 | | PPM1K | | RAB32 | | SETD9 | | SLC25A36 | |
| MIEF1 | MRPL36 | | MTERF | | NDUFAF5 | | NT5DC3 | | PDE12 | | PPTC7 | | RAB35 | | SFXN1 | | SLC25A37 | |
| MINOS1 | MRPL42 | | MTERFD2 | | NDUFAF7 | | NUBPL | | PDHB | | PPWD1 | | RBFA | | SFXN5 | | SLC25A40 | |
| MMAB | MRPL45 | | MTG1 | | NDUFB4 | | NUDT5 | | PDHX | | PRDX6 | | RCN2 | | SHMT1 | | SLC25A42 | |
| MMACHC | MRPL49 | | MTHFD1 | | NDUFB9 | | NUDT9 | | PDK1 | | PRELID2 | | RDH11 | | SIRT5 | | SLC25A43 | |
| MMADHC | MRPS10 | | MTHFD2 | | NDUFC1 | | OCIAD2 | | PDK3 | | PREPL | | RDH13 | | SLC16A1 | | SLC25A45 | |
| MOCS1 | MRPS14 | | MTIF2 | | NDUFC2 | | OGG1 | | PDK4 | | PRKG1 | | RHOT1 | | SLC16A7 | | SLC25A5 | |
| MPC2 | MRPS18B | | MTO1 | | NDUFS1 | | OPA3 | | PDP1 | | PRSS35 | | RMDN3 | | SLC25A11 | | SLC25A51 | |

|  |  |  |  |  |
| --- | --- | --- | --- | --- |
| TFAM | TOMM70A | KARS | LETMD1 | SLC25A53 |
| TG | TRIAP1 | KIAA0100 | LONP2 | SLC30A6 |
| TIMM10 | TRUB2 | KIAA0141 | LYRM2 | SLC30A9 |
| TIMM10B | TST | KIAA0391 | LYRM4 | SLC37A4 |
| TIMM22 | TTC19 | KIF1B | LYRM5 | SLMO2 |
| TIMM50 | TXN2 | KMO | LYRM7 | SND1 |
| TIMM8B | TXNRD1 | L2HGDH | LYRM9 | SOD1 |
| TIMM9 | UNG | LAMC1 | MAOA | SOD2 |
| TK2 | UQCR11 | LETM2 | MARS2 | SPRYD4 |
| TMBIM4 | UQCRB | KARS | TCAIM | SPTLC2 |
| TMEM143 | UQCRFS1 | KIAA0100 | TCHP | STOML1 |
| TMEM186 | UQCRQ | KIAA0141 | TDRKH | STX17 |
| TMEM236 | VDAC1 | KIAA0391 |  | SUCLA2 |
| TMEM65 | WARS2 | KIF1B |  | SUCLG1 |
| TMLHE | WDR81 | KMO |  | SYNJ2BP |
| TOMM20 | XPNPEP3 | L2HGDH |  |  |
| TOMM22 | YME1L1 | LAMC1 |  |  |
| TOMM40 | ZADH2 | LETM2 |  |  |
| TOMM6 |  |  |  |  |
